# Collagen Extraction from Animal Skin

**DOI:** 10.3390/biology11060905

**Published:** 2022-06-13

**Authors:** Andrea Marie E. Matinong, Yusuf Chisti, Kim L. Pickering, Richard G. Haverkamp

**Affiliations:** 1 School of Food and Advanced Technology, Massey University, Palmerston North 4410, New Zealand; a.matinong@massey.ac.nz (A.M.E.M.); ychisti@hotmail.com (Y.C.); 2 School of Engineering, University of Waikato, Hamilton 3240, New Zealand; klp@waikato.ac.nz

**Keywords:** collagen, extraction, skin

## Abstract

**Simple Summary:**

Collagen is useful in many applications including cosmetics, medicine, yarn production and packaging. Collagen can be recovered from skins of animals raised for meat. Here, we review methods for the extraction and purification of collagen from animal skins.

**Abstract:**

Collagen is the most abundant structural protein in animals. It is the major component of skin. It finds uses in cosmetics, medicine, yarn production and packaging. This paper reviews the extraction of collagen from hides of most consumed animals for meat with the focus on literature published since 2000. The different pretreatment and extraction techniques that have been investigated for producing collagen from animal skins are reviewed. Pretreatment by enzymatic, acid or alkaline methods have been used. Extraction by chemical hydrolysis, salt solubilization, enzymatic hydrolysis, ultrasound assisted extraction and other methods are described. Post-extraction purification methods are also explained. This compilation will be useful for anyone wishing to use collagen as a resource and wanting to further improve the extraction and purification methods.

## 1. Introduction

**Figure 1:**
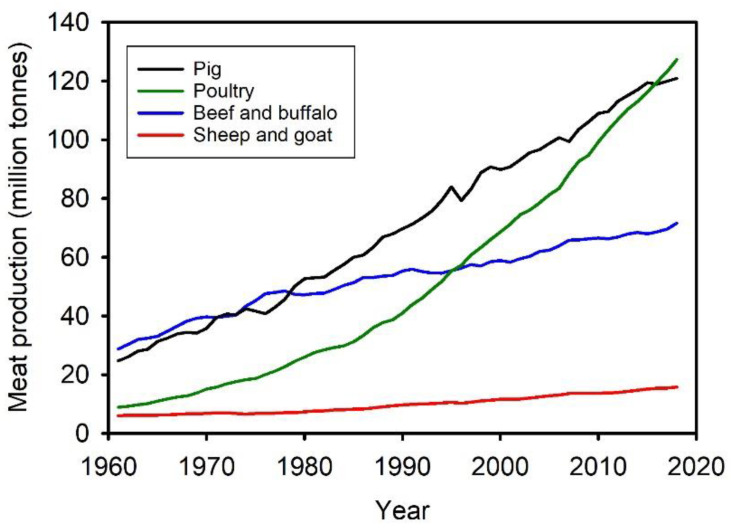
World meat production (1961–2020). Data from Ritchie and Roser [7,9].

Animal hides have been used in diverse applications since prehistoric times. Hide or skin contributes between 3% and 12% (Table 1) to the weight of a live mammal and may reach up to 20% in poultry (Table 1) [1]. Hides are mostly a by-product of meat production [2,3,4]. As meat for human consumption is mostly bovine (beef), porcine (pork), ovine (lamb), hircine (goat) and galline (chicken) [5,6,7], hides of these animals are most readily available. Hides are generally a low value by-product of meat production, and for some animals, for example sheep, typically go to landfill. Hides have the potential to be used for adding value to meat production operations [8]. Increasing global consumption of meat (Figure 1) inevitably means an increasing supply of hides [7]. These hides need to be better utilized also to minimize environmental impact [7,9].

Among other applications, hides are used: as a source of the protein keratin for extraction and incorporation in animal feed [10], fertilizers [11], cosmetics [12] and food packaging [13]. In addition, gelatine, a food additive and packaging material [14,15], is produced from hides. Furthermore, hides are a major source of collagen for use in cosmetics [16,17], medicine [18], yarn production [19] and packaging [16,20].

This paper reviews the extraction of collagen from hides of the most commonly consumed animals as listed in Table 1. The focus is on the literature published since 2000.

**Table 1:** Skin as percentage of total body mass.

Animal	Skin (% *wt*/*wt*)	Reference
Cattle	5.1–8.5	[1] [1] [1]
Sheep	11.0–11.7	
Pig	3.0–8.0	
Goat	~9.0	[21]
Chicken	8.0–20.0	[22]

## 2. Collagen

**Figure 2:**
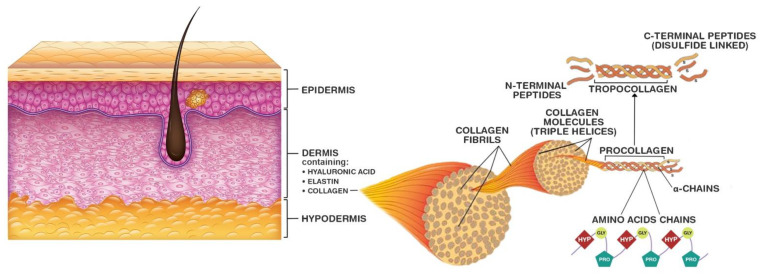
Skin-collagen-molecular structure. Adapted with permissions from: (1) Tang et al., Journal of Voice, published by Elsevier Inc. (Amsterdam, The Netherlands), 2017; (2) Reilly and Lozano, Plastic and Aesthetic Research, published by OAE Publishing Inc., 2021 (©2021 MINERVA Research Labs Ltd. (106 New Bond St. London, UK) all rights reserved) [29,30].

Collagen is the most abundant structural protein in all animals [23]. It is a component of the extracellular matrix (ECM) of various connective tissues such as skin, bones, cartilage and tendons [24]. Naturally synthesized collagen molecules consist of three long helicoidal chains of amino acid residues with nonhelical terminals at both ends [24] (Figure 2). At least 46 unique polypeptide chains have been found in collagens of various animals [23].

Collagen chains most commonly consist of the repeating motif Gly-X-Y where Gly is the amino acid glycine while X and Y are generally the amino acids proline and 4-hydroxyproline, respectively [23]. This motif is distinct from the other ECM components [23,25,26]. The α-chains of different types of collagens vary in composition, depending on the frequency of repetition and the length of the segment containing the Gly-X-Y motif, with or without interruptions, and the amino acid residues that occur in positions X and Y [27,28].

The arrangement of the polypeptide chains and the variation of the terminals gives distinction to the types of collagens, both fibrillar and non-fibrillar. Collagen types vary in conformations resulting in different lengths of helices and distributions of non-helical segments. These criteria are used to group collagens into several groups. General groups include fibrillar collagens, FACIT (fibril associated collagens with interrupted triple helices), FACIT-like collagen, basement membrane collagen, beaded filament collagen, transmembrane collagen, short-chain collagen, and unclassified collagen [31,32]. At least 29 types of collagens are currently recognized [27,31]. Fibrillar collagens are the most abundant ECM proteins in vertebrates, providing stability, connectivity and form to tissues and organs [32,33]. The most abundant fibrillar collagen in most tissues is type I collagen [34]. It is primarily present in fibril surfaces and connective tissues of the skin and bones [35]. Collagen type I has a rod-like structure made of three helically coiled chains. It has a molecular weight of approximately 300 kDa, with a length of 280 nm, a diameter of 1.4 nm and contains about 1020 amino acid residues per chain [27,36,37].

**Figure 3:**
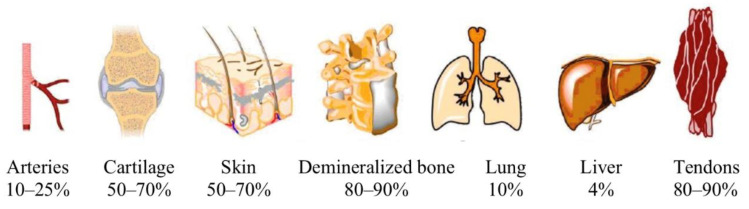
Collagen content of different body parts of animals. Reproduced with permission from Jafari et al., Polymers, 2020; 12(10):2230. ©2020 MDPI [24].

Collagen is widespread in mammal tissues (Figure 3) [24,38] and therefore can be sourced from various abattoir by-products including hides [38]. The type of collagen depends on the tissue type. Fibrillar collagens associated with different tissue types are shown in Table 2 [32,39]. Biocompatibility, biodegradability, and low antigenicity of collagen make it an attractive material for various applications [16].

### Differences in Skin of Different Animals

There are significant differences in skins of different animals. These include both the composition and the structure of the skin. For example, skins of chicken [41] and sheep are high in fat whereas beef and goat have skins with a lower fat content [42]. There may be differences in the composition of the collagen, specifically the hydroxyproline content. The arrangement of the collagen fibrils also varies markedly among species, with a higher alignment of the fibrils in species such as goat, buffalo and cattle than in sheep and pig [43,44]. Within a species, different breeds may show differences in skin structure and composition. In cattle, ‘looseness’ is a known problem in leather processing which results from differences in collagen fibril alignment [45]. These compositional and collagen structural differences have to potential to affect ease of extraction.

## 3. Collagen Extraction Process

The collagen extraction process depends on the source material. The aim is to remove all the non-collagenous matter and recover collagen as the final product. The recovery process typically involves pretreatment of the source tissue, collagen extraction, and further purification [46]. For recovery from skin, the pretreatment steps are generally preceded by washing the skin by soaking in cold water for a few days while replacing the water every few hours. This is followed by cutting the skin into more manageable pieces. Typically, skin is cut into small 1 cm^2^ pieces [47].

### 3.1. Pretreatment

Pretreatment is intended to break the covalent intermolecular crosslinks between collagen molecules. In vivo, these crosslinks facilitate collagen’s structural and mechanical functions in tissues and organs [33]. These crosslinks breakdown very slowly even in boiling water. Various mild chemical treatments are used for breaking the covalent links [37]. Dilute acids and alkalis are typically used for partial hydrolysis of the collagen. This cleaves the crosslinks, while leaving the collagen chains intact [48]. Certain enzymes may also be used for the pretreatment step [37].

#### 3.1.1. Acid Pretreatment

For acid pretreatment, washed and chopped skin pieces are immersed in dilute acid at a controlled temperature. The acid permeates the skin causing it to swell to between two- to three-times its initial volume and hydrolyzes the crosslinks [38,49,50]. Acid pretreatment is suitable for relatively fragile skins [50] that have a lower degree of fibre intertwinement, such as porcine and fish skins [38].

#### 3.1.2. Alkaline Pretreatment

Dilute alkalis such as sodium hydroxide (NaOH) and calcium hydroxide (Ca(OH)_2_) are used for pretreament. The duration of pretreatment depends on the thickness of the material being treated [49]. Alkalis are particularly effective in extracting collagen from thick and hard materials [8,38]. Pretreatment with NaOH may take from a few days to weeks to complete [48]. The lengthy treatment notwithstanding, alkaline treatment with NaOH is often preferred as it swells the skin substantially ensuring diffusion of the alkali deep into the tissue matrix [51]. Alkalis hydrolyze the unwanted non-collagenous proteins, lipids, pigments, and other organic material [52]. The treatment conditions such as the temperature, duration and the concentration of alkali significantly impact the efficacy of removal of the unwanted non-collagen material [53]. A NaOH concentration ranging from 0.05 to 0.10 kmol m^−3^ is sufficient for the pretreatment [38]. Within this concentration range, much of the acid soluble collagen and its native structure are retained in the tissue so long as the treatment temperature is between 4 and 20 °C. A high concentration of NaOH (e.g., ≥0.2 kmol m^−3^) can lead to a substantial loss of acid soluble collagen. A 0.5 kmol m^−3^ NaOH concentration may degrade the native structure of acid soluble collagen [38].

#### 3.1.3. Skin-Specific Pretreatments

The source, or the nature of the skin, may necessitate skin-specific pretreatments including soaking, fleshing, dehairing, and cutting [46]. For example, skins with fur and feathers may require different pretreatments. Mechanical slicing may be used to remove some of the fat adhering to the hide [20,54,55,56,57,58]. Alcohols such as butyl alcohol may be used to solubilize fat and pigments from chicken [55,56] and bovine skins [52,53,59,60]. Other pretreatment steps may use heating [61,62], detergents [63], solvents such as petroleum ether [64,65], hexane [66], and hot water rinses [67]. A demineralization step may involve the use of the chelating agents such as ethylene diamine tetra acetic acid (EDTA) [68,69].

### 3.2. Extraction

Conventional extraction methods have typically relied on chemical hydrolysis using acid, alkali or salt solubilization. In some cases, the chemical extraction may be aided by ultrasound or microwaves, and enzymes [70]. Extraction temperature is controlled at a relatively low value (4 °C) to minimize degradation of collagen [52].Extraction methods can be tailored depending on the desired yield and the properties of the final product. Collagen properties such as the average length of the polypeptide chains, the solubility, the solution viscosity, thermal stability, emulsifying capacity, and water retention are affected by the specifics of the extraction method [57,70]. In addition, the specific processing conditions, including the pretreatment used, the storage conditions for the hides, and their specifics (animal type, age and gender) influence the quality of the extracted collagen [27]. For example, breed and age of poultry affects the characteristics of the collage extracted [56,61]. Differences in collagen structure, for example the hydroxyproline content, can also affect the extraction process.

#### 3.2.1. Acid Hydrolysis

Collagen is most commonly extracted from skins by a hydrolysis treatment involving either acids or alkalis. Both inorganic and organic acids can effectively cleave bonds in collagen to enable extraction of the fibrils [71]. Under acidic conditions the collagen molecules have a net positive change and the resulting electrostatic repulsive force between them facilitates molecular separation [52]. The organic acids commonly used are acetic, chloroacetic, citric and lactic acids. Acetic acid has been widely reported for collagen extraction [16,48,50,54,55,56,59,71,72]. Among inorganic acids, the commonly used ones include hydrochloric, sulfuric and nitric acids [8,38,72,73]. Organic acids are apparently more effective for cleaving collagen crosslinks, and result in higher extraction yield [16,72,74] compared to mineral acids. Organic acids also solubilize non-crosslinked collagens [73]. For extraction with acetic acid, a typical concentration is 0.5 kmol m^−3^ with contact time of 2472 h and continuous stirring [75].

#### 3.2.2. Alkali Hydrolysis

Alkaline hydrolysis may also be used to extract collagen most commonly with aqueous sodium hydroxide or potassium hydroxide [52] although calcium oxide, calcium hydroxide, and sodium carbonate can also be used as extractants [76]. Alkalis have a tendency to hydrolyze collagen fibrils [71] and the amino acids cysteine, histidine, serine, and threonine may be destroyed in the process [52,76]. The use of alkali hydrolysis appears to be mostly confined to extracting collagen from leather processing waste [71,77], however, a collagen-based flame retardant has been produced from collagen obtained by alkaline extraction of otherwise untreated cattle skin [78].

#### 3.2.3. Salt Solubilization

Solubilization with salts is used less commonly [38,52]. Solutions of neutral salts are effective in solubilizing collagen and are commonly used in extraction. Examples of the salts used are citrates, phosphates, sodium chloride, and Tris-HCl [38]. Collagen type I dissolves at salt concentrations of <1.0 kmol m^−3^ but precipitates at concentrations exceeding 1.0 kmol m^−3^ [71]. This limitation on salt concentration requires careful control when extracting with salts compared to alkaline and acid hydrolysis [70].

#### 3.2.4. Enzyme Hydrolysis

Enzyme hydrolysis has been developed to address some of the shortcomings of the more traditional methods [70]. Enzyme hydrolysis may be used in combination with some of the traditional chemical methods [71]. Enzyme hydrolysis offers better reaction selectivity and is less damaging to collagen. Therefore, it has the potential to maximize collagen yield and purity of the extracted product [71]. Enzymes tend to be much more expensive than acids, alkalis and salts, but they can be used under mild reaction conditions. Compared to chemicals, enzymatic treatment is less corrosive to processing equipment, consumes less energy, produces less waste, allows better control of degree of hydrolysis, and the final hydrolysate has a lower salt content [70,71]. Hybrid methods involving chemicals as well as enzymes have been reported [71].

Proteolytic enzymes are used in collagen extraction. These enzymes may be of animal origin (e.g., trypsin, pepsin), plant origin (e.g., bromelain, papain, ficin), or microbially produced single or mixed enzymes (e.g., collagenase, proteinase K, Alcalase^®^ (Novozymes, Bagsværd, Denmark), Nutrase^®^ (Nutrex, Hoogbuul, Belgium), Flavourzyme^®^ (Novozymes, Bagsværd, Denmark), and Protamex^®^ (Novozymes, Bagsværd, Denmark)). Pepsin from animal sources is most widely used in collagen extraction [8,16,52]. Pepsin, trypsin and papain only act on the non-helix portion of the peptide chain of collagen (the ends) and leave the structurally important helical portion intact [71]. Additionally, the extracted pepsin-soluble collagen generally has a higher purity as the non-collagenous proteins are efficiently hydrolyzed by the enzyme. Pepsin treatment also increases acid solubility of collagen, which increases the extraction efficiency if used in combination with acid extraction. The yield of pepsin soluble collagen is significantly affected by the degree of crosslinking in the telopeptide region of the peptides [70]. The plant enzymes such as papain, also allow good control of the extent of hydrolysis of the protein substrate [38].

A two-step extraction process without a preceding treatment has been reported for recovery of collagen from sheepskin; an enzymatic step was used first and this was followed by acid hydrolysis [79]. Another hybrid extraction procedure using acetic acid in combination with pepsin has been described by several authors [55,56,59,60,62,68,80,81,82,83,84].

#### 3.2.5. Ultrasound-Assisted Extraction

Application of ultrasound (frequency of ≥20 kHz) during collagen extraction improves yield and reduces the time required for extraction [85]. Ultrasound generates intense turbulence in a liquid resulting in enhancement of mass transfer and rates of chemical reactions [38,86]. In ultrasound-assisted acid extraction of collagen from sea bass skin the structure of the extracted collagen remained undamaged [87], although sufficiently intense ultrasonic treatment has the potential to damage protein structure. The collagen yield is influenced by the amplitude of ultrasound and the duration of the treatment. A higher amplitude has been found to shorten extraction time and enhance yield [70]. Ultrasound assistance has been found to damage collagen structure during alkali treatment because the alkali may weaken hydrogen bonds and break parts of covalent bonds in collagen which then facilitates damage by ultrasound [64]. However, with enzyme treatment, the yield and collagen purity are improved using ultrasound and the extraction time is reduced. In ultrasound-mediated pepsin-based extraction of collagen from cattle tendon, extraction efficiency and the collagen quality were enhanced compared to a process involving only the enzyme [75]. Use of sonication in combination with enzymes increases collagen yield and shortens extraction time [88]. Ultrasound treatment is generally simple, reduces the need for corrosive chemicals, and can be carried out economically and safely. Damage to the structure of collagen has been observed in some cases [64]. Damage may be minimized by optimization of the treatment (ultrasound amplitude, duration, concentration of enzyme or other chemical, temperature) for the specific collagen source [38,52,88].

#### 3.2.6. Other Extraction Methods

Microwave-assisted. Microwave-assisted extraction has been extensively described. Microwaves, a form of electromagnetic radiation used in microwave cooking, disrupt cell and tissue structure [89,90], facilitating extraction. Microwaves penetrate deeply in tissue [90]. Microwave assistance has been found to speedup acid and enzyme action compared to equivalent treatments without the microwaves [89].

Mechanical agitation. Tissue solubilization processes involving acids, alkalis and enzymes are generally enhanced by mechanical agitation. Agitation improves hydrolysis by improving mass transfer of enzymes and chemicals into tissue. Thus, it shortens extraction time or enhances collagen yield in a given extraction time [91]. Agitation-assisted hydrolysis for collagen recovery from jellyfish tissue was shown to enhance yield by at least 5-fold compared to hydrolysis under static conditions using acid, or enzymes alone [92]. Similar observations were reported by others [68].

#### 3.2.7. Precipitation of Solubilized Collagen

Once the collagen has been solubilized by the methods described above, it needs to be precipitated from solution. Salts are most often used to achieve this, with a salt concentration exceeding 1.0 kmol m^−3^. This treatment has been used for collagen from chicken skin [62,63,80,93], sheep and goatskin [54,55,68,81], pigskin [82,94], and cattle skin [59,95].

### 3.3. Post Extraction: Purification

In addition to collagen, a crude collagen extract typically contains neutral salts and non-collagen proteins [46]. Collagen can be purified from this extract to obtain collagen fractions with different molecular weights. Purification processes use multiple steps [96] which may include filtration and centrifugation [85]. Target protein, or unwanted proteins, may be precipitated from solution by “salting out” (adding a high concentration of salt to extract).

Th salting out of collagen, or other proteins, in a crude extract is influenced by several factors, including temperature, pH, the ionic strength, the salt used, and the specifics of the proteins involved. Generally, collagen is less soluble than the contaminating proteins and may be precipitated by salting out. Salting out conditions must be selected to minimize coprecipitation of the other proteins.

Lyotropic series can be used as a guide in selecting salts for salting-out [46,97]. Both anions and cations in a salt are ranked in the order of their salting-out ability [46]. Anions are more efficient in salting out than cations. For anions, the salting out efficiency decreases in the following order: F^−^ ≈ ${\mathrm{SO}}_{4}^{2-}$ > ${\mathrm{HPO}}_{4}^{2-}$ > CHCOO^−^ > Cl^−^ > ${\mathrm{NO}}_{3}^{-}$ > Br^−^ > ${\mathrm{ClO}}_{3}^{-}$ > I^−^ > ${\mathrm{ClO}}_{4}^{-}$ > SCN^−^. For cations, the salting out performance decreases in the following order: ${\mathrm{NH}}_{4}^{+}$ > K^+^ > Na^+^ > Li^+^ > Mg^2+^ > Ca^2+^. Neutral salts produced by combining a strong anion and a cation from the above series, have a strong ability to precipitate collagen from a solution. Sodium chloride is a neutral salt with a strong precipitating effect. Although ammonium sulfate is generally the best for salting out proteins, it is not a neutral salt and its constituent ions are often not acceptable in products intended for biomedical applications [46]. Neutral salts have the advantage of not significantly affecting the pH of a solution, as pH is also a factor in slating out.

For precipitation of collagen, a suitable mass of a neutral salt is added to a specific volume of the collagen containing solution [46]. The pH is adjusted to 7 by adding sodium hydroxide. The resulting solution is allowed to stand for 4–12 h. The collagen precipitate is collected by centrifugation. The recovered precipitate may be dissolved and subjected to a second round of salting-out. This procedure may be repeated twice or more for further purification [46]. Refrigerated centrifuges (4 °C) are typically used in recovering the precipitate.

The precipitated collagen contains a large amount of salt. This is removed by dialysis. Precipitated collagen is placed in dialysis bag and dialysed against an acidic solution or deionised water. During dialysis, the dialysate is periodically changed to increase the rate of ion migration [96]. Various dialysis schemes have been reported [38,59]. A single or multiple dialysis stages may be used. Dialysis may take 4 to 10 days to sufficiently remove the salts [46]. This slow step contributes nearly 50% to the total time required for the entire collage extraction process [46].

Purification methods may be customized to recover collagen of a certain molecular weight range [70]. Various types of membrane filtrations and chromatographic separations can be used to finetune the molecular weight of the collagen product [96,98].

## 4. Specific Extraction Methods

The collagen extraction methods that have been described in the literature are summarized in Table 3.

**Figure 4:**
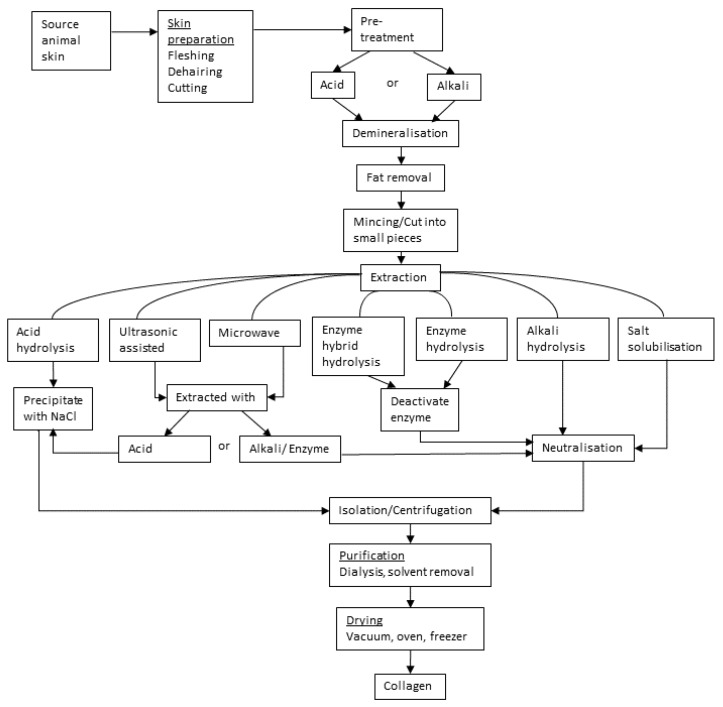
Overview of processing options for extraction of collagen from skin (based on Table 3).

The pretreatments listed in Table 3 usually have the purpose of removing some of the non-collagenous material but also serve the important function of “opening up” the skin to enable easier penetration of the reagents that are used to solubilize collagen. In some cases, the pretreatment might include similar chemistry to the subsequent treatment, but generally with lower concentrations of reagents. The main options described in Table 3 may be summarized pictorially, as in Figure 4.

### Future Considerations

While a range of methods for extracting collagen from animal skins has been described, these do not cover the skin from all commercially reared meat animals. Skin from some animal species have not been investigated in detail, notably sheep and goats, which represents an opportunity for further study and exploitation. The nature of the collagen resulting from the extraction processes has often not been well characterised. For example, do the collagen fibrils remain intact, or do they reform spontaneously, or are they degraded partially? Also what is the impact of these factors on the mechanical properties of materials manufactured using the extracted collagen? This is an area where further knowledge would be useful.

## 5. Conclusions

A wide variety of collagen extraction and separation methods have been described for animal skins from a number of meat-producing species. These have been categorized and summarized here showing the options available to produce a collagen extract. Multiple choices are clearly available for the commercial production of collagen from various animal skins.

## Figures and Tables

**Table 2:** Collagen types, compositions and sources (based on [39,40]).

Type	Molecule	Source
I	${\left[\alpha 1\left(I\right)\right]}_{2}\left[\alpha 2\left(I\right)\right]$	Skin, tendon, bone, ligaments, interstitial tissues
II	${\left[\alpha 1\left(II\right)\right]}_{3}$	Intervertebral disc, cartilage, vitreous humor
III	${\left[\alpha 1\left(III\right)\right]}_{3}$	Cardiovascular vessel, uterus, skin, muscle
V	[α1(V)][α2(V)][α3(V)]	Similar to type I, also cell cultures, fetal tissues; associates with type I
XI	[α1(XI)][α2(XI)][α3(XI)]	Cartilage, in vertebral cartilage and bone enamel

**Table 3:** Pretreatment, extraction and isolation methods for collagen production from skin.

Pretreatment	Extraction and Isolation	Reference
Chicken		
Hot-water bath (40 and 60 °C, 1 h)	Multi-step extraction (4 °C): (1) Protease inhibitors solution: 1 kmol m^−3^ NaCl and 50 × 10^−^^3^ kmol m^−3^ Tris-HCl with PhCH_2_SO_2_F (1 × 10^−^^3^ kmol m^−3^), MalNEt (10 × 10^−^^3^ kmol m^−3^) and EDTA (20 × 10^−^^3^ kmol m^−3^) for 24 h; (2) Ethylene diamine dihydrochloride, 24 h; (3) 0.5 kmol m^−3^ acetic acid; (4) 0.5 kmol m^−3^ acetic acid with pepsin (1 mg/mL). Followed by precipitation steps: Ammonium sulfate (25% saturation) for precipitation in between extraction rounds. NaCl (crystals) for collagen type-specific precipitation.	[62]
Non-collagen removal (0.1 kmol m^−3^ NaOH, 6 h); fat removal (10% *v*/*v* butyl alcohol, 24 h, 4 °C)	Acid hydrolysis (0.5 kmol m^−3^ acetic acid, 42 h, 4 °C); precipitation (2 kmol m^−3^ NaCl); dialysis (water); centrifugation	[93]
Non-collagen removal (0.1 kmol m^−3^ NaOH, 6 h); fat removal (4% detergent, Triton X-100 and 5% KCl, 12 h)	Acid hydrolysis (0.5 kmol m^−3^ acetic acid, 3 days at <10 °C); precipitation (2.5 kmol m^−3^ NaCl and 0.05 kmol m^−3^ Tris-(hydroxymethyl)-aminomethane); centrifugation	[63]
Fat and pigment removal (centrifugation)	Acid hydrolysis (0.5 kmol m^−3^ acetic acid, 72 h at 4 °C); centrifugation; dialysis (distilled water)	[61]
Non-collagen removal (0.1 kmol m^−3^ NaOH, 24 h at 4 °C); fat removal (10% butyl alcohol, 24 h at 4 °C)	Acid hydrolysis (0.5 kmol m^−3^ acetic acid, 24 h at 4 °C); vacuum (varies depending on breed)	[99]
Fat and pigment removal (centrifugation); non-collagen removal (2 kmol m^−3^ NaOH, 12 h)	Acid hydrolysis (0.5 kmol m^−3^ acetic acid, 24 h at 4 °C); precipitation (0.9 kmol m^−3^ NaCl); for acid insoluble collagen: (1) heat soluble collagen (95 °C), (2) enzyme hydrolysis (1% *w*/*w* pepsin; centrifugation; dialysis (deionized water)	[80]
Sheep		
Conducted at 4 °C. Non-collagen removal (0.1 kmol m^−3^ NaOH, 6 h); demineralize (0.5 kmol m^−3^ EDTA-2Na, 48 h)	Acid hydrolysis (0.5 kmol m^−3^ acetic acid, 3 h at 20 °C); enzyme addition (pepsin 1 g L^−1^, 48 h); precipitation (2.6 kmol m^−3^ NaCl); centrifugation; second hydrolysis (1 kmol m^−3^ NaCO_3_, trypsin 1:50 *w*/*v* at 60 °C from 10 min to 4 h)	[68]
Wash; dehair (deionized water)	Acid-enzyme hybrid (0.5 kmol m^−3^ acetic acid, 0.01 and 0.001 g g^−1^ trypsin, at 20 and 35 °C, pH 7 and 9 for 30–360 min); filtration and centrifugation	[79]
Goat		
Non-collagen removal (0.1 kmol m^−3^ NaOH, 0–48 h at 4 °C)	Conducted at 4 °C. Acid-enzyme hybrid (0.5 kmol m^−3^ acetic acid with 0.1% *w*/*v* pepsin, 24 h); precipitation (2.6 kmol m^−3^ NaCl, 12 h); centrifugation (4500× *g*, 30 min)	[55]
Non-collagen removal (0.1 kmol m^−3^ NaOH, 24 h at 4 °C)	Acid hydrolysis (0.5 kmol m^−3^ acetic acid 24–72 h); precipitation (2.6 kmol m^−3^ NaCl); centrifugation (7000× *g*); redissolution and dialysis (acetic acid)	[54]
Non-collagen removal (0.1 kmol m^−3^ NaOH, 0–48 h at 4 °C)	Acid-enzyme hybrid (0.5 kmol m^−3^ acetic acid with 0.1% pepsin, 24–72h at 38 °C); precipitation (2.6 kmol m^−3^ NaCl); centrifugation (7000× *g*, 30 min at 4 °C); redissolution and dialysis (acetic acid)	[81]
No pretreatment detailed	Enzyme hydrolysis (1 g pepsin in 100 mL buffer (pH 2.0) at 37 °C for 15 min); second enzyme addition (0.1 U pepsin, 1–120 min); neutralization (1 kmol m^−3^ NaOH); centrifugation (1000× *g*, 15 min)	[100]
Pig		
Degrease in ultrasonic bath (75% sodium dodecylbenzene (SDBS)), skin to SDBS 1:2.5 volume ratio at 25 °C, 120 W); non-collagen removal (1% NaCl for 6 h)	Conducted at 4 °C. Acid-enzyme hybrid (2000 U g^−1^ pepsin in 0.5 kmol m^−3^ acetic acid, 18 h); precipitation (NaCl, 8–12 h); centrifugation; redissolution and dialysis (both acetic acid, and water for second dialysis)	[56]
No pretreatment detailed	Alkaline treatment (3–7% NaOH in 6% NaCl solution, 24 h); neutral salt wash (NaCl); neutralization (7% acetic acid); vacuum freeze dry (75–90% moisture removal)	[94]
Fat removal (1:3 *w*/*v* petroleum ether at 30 °C, 1 h)	Enzyme hydrolysis (microfluidizer) (2400 U g^−1^ pepsin (pH 7, 50 °C) and 3000 U g^−1^ Alcalase (pH 8.5, 60 °C)); centrifugation (10,000× *g*, 4 °C, 20 min); dialysis and freeze dry	[65]
Fat removal (hexane, 60 g skin per 400 mL); drying (24 h at 60 °C, −76 mm Hg)	Hydrolysis with rotary evaporator (125 g L^−1^ of acid with pH 3 or alkali with pH 12 at 60 °C for 1 h at rotary speed scale 6); centrifuge (8000× *g*, 10 min); freeze dry	[66]
Fat removal (petroleum ether); non-collagen removal (1% *w*/*v* NaCl, 6 h); alkali pretreatment (2% g g^−1^ NaOH with 2:5 g skin mL^−1^ solution ratio, 1 h); ultrasound (in alkali solution, at 25 kHz at 290 W, 40 min)	Maintain basic pH (0.1 kmol m^−3^ phosphate buffer with pH 8.0); enzyme hydrolysis (Alcalase 1:100 *w*/*v* at 55 °C, varying hydrolysis times); centrifugation	[64]
Fat removal (10% Na_2_CO_3_; hot water bath 45 °C)	pH adjustment (pH 8); enzyme hydrolysis (2 h at 40 °C, enzyme not mentioned); filtration and centrifugation; freeze dry (supernatant)	[20]
In a rotating drum: Wash (0.3% peregal at 30 °C for 3 h); fat removal (manual defleshing; 300% float with 2.5% Na_2_CO_3_ and 0.5% peregal at 30 °C for 3 h); dehair (2.5% trypsin (250 µ mg^−1^) coated on flesh side at 25 °C overnight)	Alkali-enzyme hydrolysis (not detailed); freeze dry (−5 °C for 5 h) (stirred at 4 °C); acid hydrolysis (2 kmol m^−3^ acetic acid with 1:50 *w*/*v*, 6 h) centrifugation (20,000× *g*, 30 min); neutralization (NaOH addition until pH 7.5); precipitation (1.5 kmol m^−3^ NaCl, refrigerated desiccator for 12 h); centrifugation; dialysis(water until neutral pH, 30 kDa molecular weight cutoff membrane; freeze dry; acid-enzyme hybrid (0.5 kmol m^−3^ acetic acid solution containing 1.5% pepsin with 1:20 *w*/*v* ratio for 3 days); centrifugation and precipitation; dialysis (2×); freeze dry	[82] Based on [101] and patent CN1569260
Fat and flesh removal (mechanical removal); wash (phosphate-buffered saline)	Decellularized collagen: Supercritical CO_2_ vessel system (75% ethanol, 30–50 °C and 200–350 bar for 40min); neutralization (0.1–1 kmol m^−3^ NaOH); drying and sterilization (γ-irradiation 25 kGy). Atelocollagen: Milling (freeze milled with liquid nitrogen, 50–200 µm); acid and enzyme digestion (0.01 kmol m^−3^ HCl containing 1 g L^−1^ pepsin, stirred for 16–18 h at 25 °C); filtration (1) 0.1–0.4 µm, (2) >150 kDa, (3) 0.2 µm; fibrillogenesis (3 mg mL^−1^ acidic atelocollagen solution with 0.2 kmol m^−3^ phosphate buffer at a ratio of 9:1 *v*/*v*); centrifugation (7000× *g*, 30 min at 4 °C); freeze dry (precipitate)	[102]
Hot water bath (1:9 *w*/*v* at 60 °C, 30 min)	Acid/alkali-enzyme hydrolysis ((1) 762 U g^−1^ pepsin, (2) trypsin and (3) Alcalase with 1 kmol m^−3^ HCl or NaOH at their respective optimal temperature and pH, 4 h); inactivate enzyme, neutralization, filtration, dialysis and freeze dry	[103]
Alkali treatment (0.1 kmol m^−3^ NaOH at 1:5 volume ratio, 3 days)	Acid hydrolysis and filtration (0.5 kmol m^−3^ acetic acid for 3 days, 2×. Filtrate was collected separately); freeze and precipitated (frozen 24 h; added in 0.9 kmol m^−3^ NaCl for 12 h). Centrifugation; dissolution and dialysis (acetic acid; dialysis at 1:10 for 2 days); freeze dry	[58]
Hot water bath (90 °C, 1 min)	Control mixture (5:100 volume ratio skin to solution (water)); enzyme hydrolysis (1) Alcalase, (2) Flavorzyme, (3) Neutrase, (4) bromeline, (5) Protamex, (6) papain-at 1:100 enzyme substrate ratio with hydrolysis times 1–24 h at optimal temperature; inactivation and cooling; centrifugation (4000× *g*, 15 min); filtration (3 kDa, 60 psi nitrogen gas at 20 °C); freeze dry	[67]
Cattle		
No pretreatment detailed	Alkali hydrolysis (3–5% NaOH, stirred at 60 °C); filtration (filtrate collected); neutralization (acetic acid); dialysis	[78]
Neutral salt wash (10 volumes (*v*/*w*) 0.5 kmol m^−3^ NaCl for 2 h); lime treatment (dehair) (40 g L^−1^ lime suspension at 1:10 *w*/*v* for 4 days); non-collagen removal (2% NaOH at 1:10 *w*/*v* for 5 days)	Conducted at 4 °C, stirred. Acid-enzyme hybrid (0.5 kmol m^−3^ acetic acid at 1:100 *w*/*v* with pepsin addition at 1:20 *w*/*w*, 2 days); centrifugation (20,000× *g*, 20 min), precipitation (2 kmol m^−3^ NaCl, 6 h); centrifugation (15 min); dialysis (14 kDa MW, deionized water for 4 days); freeze dry	[83]
Hair removal (1) sulfide solution [104]; (2) oxidative process [105]; relime [104]; acid pretreatment (0.5% acetic acid first at pH 7.1 for 3 h and then at pH 5.4 for 4 h). Neutral salt wash (400% water containing 4.35% NaCl for 2 h, twice); lime treatment (400% water containing 0.3% lime for 2 h, thrice); acetone treatment (400% acetone, 12 h repeated 5–7 times); air dry and frozen; (pulverized)	Conducted at 4 °C. Acid hydrolysis (200–250 mg hide powder in 100 mL 0.5 kmol m^−3^ acetic acid, 3 days); centrifugation and freeze dry	[106]
No pretreatment detailed	Skin-buffer mixture (100 g L^−1^ skin to solution, 0.1 kmol m^−3^ phosphate buffer and 0.15 kmol m^−3^ NaCl with pH 7.5); alkali addition (0.001 kmol m^−3^ NaOH and 3.5g NaBH_4_, incubated for 24 h at 25 °C); acid addition (glacial acetic acid, adjusting pH to 3); centrifugation (23,000× *g* for 30 min, pellet collected); freeze dry	[95]
Hair removal (0.5 kmol m^−3^ NaOH, 24 h); non-collagen removal (3 different samples prepared: (1) 1:10 *w*/*v* 0.1 kmol m^−3^ NaOH, 6 h; (2) 20 vol 0.1 kmol m^−3^ NaOH and 0.1 kmol m^−3^ NaCl, 24 h; (3) 1:20 *w*/*v* 0.1 kmol m^−3^ NaOH and 0.1 kmol m^−3^ NaCl, 6h); fat removal/demineralization ((1) 1:10 *w*/*v* 10% butyl alcohol, 10 h; (2) 0.1 kmol m^−3^ HCl and 0.1 kmol m^−3^ NaCl 1:20 w/v, 24 h; (MAES) 0.1 kmol m^−3^ HCl and 0.1 kmol m^−3^ NaCl 1:20 *w*/*v*, 24 h)	Acid/acid-enzyme hydrolysis: (1) 30 vol. 0.5 kmol m^−3^ acetic acid, 24 h, (2) 20 vol 0.5 kmol m^−3^ acetic acid with 1% *w*/*w* pepsin, 24 h, (3) 20 vol 0.7 M acetic acid with 1% *w*/*w* pepsin, 48 h; filtration (4 mm and 250 µm filter); precipitation: (1) 2.5 kmol m^−3^ NaCl, (2) 2.5 kmol m^−3^ NaCl, (3) 2.5 kmol m^−3^ NaCl; centrifugation (various times); dialysis (14 kDa MW, acetic acid and water); freeze dry (−52 °C, 48–72 h)	[59]
Lime treatment (100 g hide in 1.5 g Na_2_S and 5 g CaO solution, soaked for 2 days); de-lime treatment (2 g NH_4_Cl and 4 mL concentrated HCl); wash (rinsed with water until neutral pH); fat removal (10 volumes 10% butanol for 24 h)	Acid hydrolysis (30 volumes 0.5 kmol m^−3^ acetic acid solution containing 1% pepsin, 2 days stirred periodically); filtration and centrifugation (2-layer cheesecloth; 10,000 g for 20 min). Precipitation (3 kmol m^−3^ NaCl); centrifugation and dissolution (acetic acid); dialysis (tris buffer)	[60]
Hot-water bath (70 °C for 15 min, dehair)	Solutions (500 g hide each sample, calcium hydroxide and acetic acid solutions were added to each beaker). Samples and concentrations (1) control, not pretreated; (2) 5% *w*/*v* Ca(OH)_2_; (3) 15% *w*/*v* Ca(OH)_2_; (4) 5% *w*/*v* Ca(OH)_2_ and 5% *v*/*v* CH_3_COOH; (5) 15% *w*/*v* Ca(OH)_2_ and 5% *v*/*v* CH_3_COOH. Hydrolyzed for 4 days); hot water bath (60–70 °C for 24 h); filtration and dehydration (filtrate collected, 60 °C oven for 24 h)	[107]
De-lime treatment (2% NH_4_Cl and 0.5% HCl, 60 min); neutralization (0.5% HCl until pH 6–7; rinsed with distilled water	Acid-enzyme hybrid (30 volumes 0.5 kmol m^−3^ acetic acid containing 1% pepsin, 48 h, 4 °C); centrifugation (10,000× *g* at 4 °C, 15 min); precipitation (3 kmol m^−3^ NaCl); centrifugation; dissolution and precipitation (0.5 kmol m^−3^ acetic acid and 0.7 kmol m^−3^ NaCl); dissolution and dialysis (0.5 kmol m^−3^ and 0.1 kmol m^−3^ acetic acid, respectively)	[84]

## Data Availability

Not applicable.
